# Strengths and weakness of neuroscientific investigations of childhood poverty: future directions

**DOI:** 10.3389/fnhum.2015.00053

**Published:** 2015-02-11

**Authors:** Sebastián J. Lipina, M. Soledad Segretin

**Affiliations:** Unidad de Neurobiología Aplicada (UNA, CEMIC-CONICET), Buenos Aires, Capital FederalArgentina

**Keywords:** childhood poverty, social inequality, brain plasticity, interventions, policy making

## Abstract

The neuroscientific study of child poverty is a topic that has only recently emerged. In comparison with previous reviews (e.g., Hackman and Farah, 2009; Lipina and Colombo, 2009; Hackman et al., 2010; Raizada and Kishiyama, 2010; Lipina and Posner, 2012), our perspective synthesizes findings, and summarizes both conceptual and methodological contributions, as well as challenges that face current neuroscientific approaches to the study of childhood poverty. The aim of this effort is to identify target areas of study that could potentially help build a basic and applied research agenda for the coming years.

## Current advances in the comprehension of brain development and plasticity in adverse developmental contexts

The study of how adverse environmental conditions (e.g., socioeconomic status (SES) or poverty) influence brain organization and reorganization during development includes different approaches. Among the most cited factors affecting development are neural plasticity, epigenetics, the influence of environmental toxins, nutrition, stress regulation, poverty modulation of cognitive and emotional processing, cognitive functioning, and health of adults with a history of childhood poverty (Hackman and Farah, 2009; Lipina and Colombo, 2009; Hackman et al., 2010; Bryck and Fisher, 2012; Miller and Chen, 2013). Specifically, current research on the timing of structural and functional development of different neural systems, the multiplicity of levels of organization, and the importance of epigenetics shows that these are important factors in the interpretation of the findings on poverty and brain development. The aim of this section is to highlight the importance of a comprehensive approach to foster the integration of the conceptual models that have been generated in the studies of brain development and plasticity to help design a new generation of research methods and proposals in the study of childhood poverty from a neuroscientific perspective.

The study of the influences of material and social deprivation on the central nervous system (CNS) has been an issue of interest in neuroscience research since the first half of the twentieth century. Early neuroscientific studies in experimental animals analyzed how exposure to complex, standard or deprived environments can modify the brain. At present, the same underlying questions still apply to the analysis of how different rearing environments (i.e., complex vs. standard) modulate brain structure and function at its many different levels (i.e., molecular, genetic, cellular, network, individual, and social-behavior levels, Hirase and Shinohara, 2014). Specifically, exposure of different species to enriched conditions, in comparison with either standard or deprived environments, has been associated with several structural changes in neurons and synapses, glial components, brain vasculature, brain cortex weight and thickness, rate of hippocampal cell neurogenesis, availability and metabolism of both neurotrophi factors and neurotransmitters in different brain areas, and neurotrophic and neurotransmitter gene expression (Hirase and Shinohara, 2014). It has been proposed that the processes involved in neuroplasticity are affected by different principles (e.g., Mohammed et al., 2002; Hirase and Shinohara, 2014). To this respect, some of the main contributions include the diversity of molecular mechanisms in different brain areas, epigenetic interactions, the role of structural consolidation, inhibitory and excitatory balance, functional competition between inputs, regulation by experience and age, influence of motivation and cognitive control, and potential for reactivation of organizational processes in adulthood (Hensch, 2004; Holtmaat and Svoboda, 2009; Bavelier et al., 2010).

Also, recent behavioral studies have shown that sensitive periods are not necessarily fixed in terms of timing, and suggest that closure of these periods is likely to result from the natural consequence of a given learning process (Michel and Tayler, 2005). In addition, they could coincide with the attainment of functional specialization in a given domain (Johnson, 2005). In the case of the neural circuits involved in complex behaviors, the closure of sensitive periods seems to depend on whether they are associated with circuits performing computations at either basic or complex levels, such as feature representation, categorization function, top-down interactions, and cross-modal reorganization (Kral, 2013). Thus, integration of the different forms of plasticity should be the focus for neuroscience research in the field of poverty and brain development aimed at establishing windows for intervention opportunities. This analysis is time-consuming and requires methodological innovations for the exploration of molecular pathways, systems and behavioral events, and phenomena simultaneously, and throughout the different stages of development (e.g., Rao et al., 2010). For instance, in experiments with infants, different tools are usually introduced to facilitate the acquisition of motor skills before the age at which these behaviors are typically observed (Smith and Thelen, 2003). These studies provide behavioral information about how experience-expectant processes can be manipulated to occur earlier than expected in a normal developmental trajectory. Therefore, measurement of neural activity that occurs before the attainment of a certain skill could allow for a better understanding of the development of the mechanisms responsible for these behaviors (e.g., Rao et al., 2010). Another example of the importance of preventive-measuring of neural activity is the study of how the hearing system is affected differently in contrasting socioeconomic contexts (Skoe et al., 2013). Hearing ability depends on different degrees of environmental noise exposure (Zhou and Merzenich, 2012), and acoustic enrichment of the environment may promote recovery of auditory cortical processing (Zhu et al., 2014).

Similarly to many areas of study on the effects of poverty on development, epigenetic analyses of early brain development in humans are in their early stages. Evidence of the modulation of epigenetic mechanisms during early development in individuals growing under different rearing conditions (e.g., deprived SES, stress exposure) has recently been incorporated into this line of research. For instance, Essex et al. (2013) examined differences in DNA methylation in adolescents for several genes (GR (NR3C1), dopamine receptor (DRD4), serotonin transporter (5HTT), brain-derived neurotrophic factor (BDNF), catechol-O-methyltransferase (COMT), and dopamine transporter (DAT1)) in relation to their parents’ reports of hardship during childhood. They found that maternal stress in infancy predicted higher methylation levels in both girls and boys, but paternal stressors in preschool predicted differences in methylation at adolescence specifically in girls. In addition, recent cumulative evidence suggests that differential susceptibility to the rearing environment may depend on variations in dopamine-related genes. For instance, Bakermans-Kranenburg and van Ijzendoorn (2011) found that children with secure attachment representations donated more money to a charity (e.g., UNICEF) in the context of an attachment story completion task, only if they had the DRD4 7-repeat allele; and that children with less efficient dopamine-related genes (D2, DRD4, DAT1) had more adaptive difficulties in negative rearing environments. More recently, these types of molecular genetic approaches are being increasingly used to examine the association between dopaminergic polymorphisms and educational achievement (e.g., Beaver et al., 2012).

Although many conceptual and methodological issues should be explored, initial epigenetic findings support the notion that epigenetic changes underlie, at least partially, the long-term impact of early experiences, and that epigenetic alterations are potentially reversible or modifiable through pharmacological or behavioral intervention (Hensch, 2004). This means that the understanding of the role of the epigenome on the behavioral modifications driven by early experiences could contribute to our understanding of the relationship between childhood poverty and brain development. However, behavioral associations with genotypes in humans should be interpreted with caution because similar experiences may produce different outcomes in different people. These potentially variable outcomes add another level of complexity to the study of how behavior is modulated by early experiences.

## Strengths of the current neuroscientific approach to study poverty

Some of the main questions currently included in the neuroscientific study of poverty focus on a number of topics already addressed by the fields of developmental psychology, cognitive psychology, and health sciences, especially those regarding the effects and mechanisms of mediation at the behavioral level of analysis (Bradley and Corwyn, 2002; Hackman and Farah, 2009; Moffitt et al., 2011; Evans et al., 2013). The aim of this section is to highlight the contributions made by neuroscientific research, that have allowed the growth and expansion of the field of poverty and brain development in recent years.

One of the areas in which these advancements have been verified is the study of stress regulation in early adverse developmental contexts. For instance, recently, the topic of stress regulation has been included in the study of poverty and cognitive development through different perspectives, such as vulnerability and environmental susceptibility (Ellis and Boyce, 2011; Hackman et al., 2012; Sheridan et al., 2013), the impact of poverty on executive functions (Blair et al., 2011), and even child development policy (Shonkoff and Bales, 2011). In all these approaches, the focus of the analytical efforts was on the analysis of the mechanisms mediating stress responses, which took into consideration a number of guiding principles that could contribute to the understanding of childhood poverty. For example, Ganzel et al. (2010) have suggested that properties (i.e., magnitude, duration and chronicity), and types (e.g., social exclusion vs. physical threat) of stressors in early adverse developmental contexts modulate the impact on neural networks involved in acute and chronic responses to stress. In this regard, future research should investigate the timing and specificity of neural development that is sensitive to stress exposure (Lupien et al., 2009).

In addition, current neuroscientific research in the area of early adverse experience on brain development has begun to incorporate concepts and methodologies derived from advances in epigenetics and the analysis of neural activation in animal and human models. Three sets of problems have started to shape the direction of the research in this area: brain plasticity in prenatal development, reactivity of the amygdala to threatening situations, and brain changes associated with adverse life experiences (Gianaros and Manuck, 2010). In concert with these issues, research programs have addressed the influence of malnutrition (Georgieff, 2007) and exposure to different types of pollutants and drugs (Hubbs-Tait et al., 2005) during pre- and post-natal brain development, with significant implications for the neuroscientific study of childhood poverty.

Despite these important advances, the neuroscientific study of human poverty, particularly child poverty, is a topic that has gained attention in the most recent decades. Since the mid-1990s, researchers have applied neurocognitive behavioral paradigms to compare the performance of children with disparate SES, and technological advances in neuroimaging have allowed for the analysis of neural networks (Hackman and Farah, 2009; Lipina and Colombo, 2009; Hackman et al., 2010; Raizada and Kishiyama, 2010; D’Angiulli et al., 2012; Lipina and Posner, 2012; Gianaros and Hackman, 2013). Specifically, tasks involving language, cognitive control and memory demands have provided evidence that suggests that these systems may be the most frequently affected by SES adverse environments.

In addition, a recent topic of interest in neuroscience addresses mechanisms of mediation of childhood poverty on cognitive development (Hackman et al., 2010; Noble et al., 2012; Lipina et al., 2013; Neville et al., 2013a), which allows the identification of potential targets for the design of interventions. In this sense, to generate changes in neurocognitive development, interventions have been introduced recently in the study of attention disorders, dyslexia, dyscalculia, executive functions, and arithmetic performance in samples of children from different SES backgrounds. In all of these studies, there has been an emphasis on the behavioral levels of analysis (e.g., Goldin et al., 2014; Segretin et al., 2014), and both neuroimaging techniques and molecular and behavioral genetics have been included in some cases (Rueda et al., 2005, 2012; Bryck and Fisher, 2012; Espinet et al., 2013; Neville et al., 2013b). All of this should help contribute with the identification and the better comprehension of the mechanisms of mediation of early adversity on brain development. For instance, Brito and Noble (2014) have proposed early linguistic environment and stress as the candidate mechanisms through which poverty influences structural (i.e., language hemisphere, hippocampus, amygdala and prefrontal cortex) and functional (i.e., language, memory, social-emotional processing, cognitive control, self-regulation) brain development, based on recent findings considering different systems and levels of organization. Thus, neuroscientific evidence generated during the last decade in the study of childhood poverty has helped to identify the early linguistic environment and the regulation of stress as two main aspects to consider in dealing with the conceptual and methodological challenges, and as future directions in the area.

In summary, the implementation of the technological advances into the study of how early adversity impacts brain development and plasticity, has allowed neuroscientists to improve the identification of mechanisms of mediation and, consequently, has opened new avenues for the innovation in the design of interventions aimed at fostering the development of different emotional, cognitive and social competences. In such a context, many lines of research that begun their development several decades ago (e.g., stress regulation) are converging in a way that seems to be useful when approaching childhood poverty from contemporary neuroscientific perspectives.

## Limitations, challenges and future directions

The advances in cognitive neuroscience research have posed several conceptual and methodological challenges in the study of childhood poverty. In terms of impacts, mediating mechanisms, hypotheses and the interpretation of data obtained by applying molecular, behavioral, and neuroimaging techniques seems to focus mainly on the comparison of performance and degree of activation rather than the identification of mediating mechanisms (Hackman and Farah, 2009; Lipina and Colombo, 2009; Hackman et al., 2010; Raizada and Kishiyama, 2010; Lipina and Posner, 2012). In addition, most of the evidence is limited to cross-sectional or short-term longitudinal designs, which present difficulty for understanding changes in the study of brain development in adverse contexts. Moreover, the consideration of sensitive periods for many processes susceptible to different socioeconomic conditions and timing of intervention requires a revision of the agendas in other disciplines addressing childhood poverty (e.g., many disciplines currently contend that the impacts of economic and social deprivation are permanent and irreversible) (D’Angiulli et al., 2012). Thus, incorporating findings that show the time-sensitivity of plasticity into research designs could contribute to revise this way of thinking about human brain development in adverse contexts.

Considering the opportunities and setbacks mentioned in the previous two sections, we propose a set of main points that require reconsideration and optimized approaches. First, we propose to increase the focus on the study of variables, factors and mechanisms that mediate the effects of poverty on different cognitive and emotional processes to complement the analysis of impacts. In this context, it is necessary to take into consideration the structural, electrophysiological, and molecular changes in brain plasticity in terms of (a) how neural operations change after adverse experience; (b) the physiological and biochemical involvement of components related to connectivity between different neural networks; (c) how experience and neuropil transformations contribute to brain functional specialization; and (d) the role of epigenetics, sensitive periods and differential susceptibility in shaping neural networks (Hackman et al., 2010; D’Angiulli et al., 2012; Hirase and Shinohara, 2014).

Second, we propose to deepen the theoretical integration of findings from human and animal models to include the consideration of epigenetic mechanisms, to overcome the limitations of only considering the behavioral or neural levels of analysis (Lipina and Colombo, 2009; Hackman et al., 2010), and to promote the simultaneous analysis of more than one level of organization.

Third, we also propose to expand the theoretical integration across all developmental and cognitive psychology, and to plan experiments applying neuroimaging techniques to promote and generate innovative hypotheses and research programs (Crone and Ridderinkhof, 2011; Gianaros and Hackman, 2013). For such a purpose, it is necessary to encourage the design of interventions and the measurement of outcomes driven by theoretical models to include the consideration of underlying mechanisms at different levels of analysis. Additionally, it should be important to use conceptual models aimed at understanding the transfer of gains across different domains beyond laboratory methodologies, such as school and work achievement (Crone and Ridderinkhof, 2011; D’Angiulli et al., 2012; Goldin et al., 2014).

Fourth, we suggest the development of innovative studies directed at analyzing plasticity of complex cognitive and emotional processes, and their respective windows of opportunities for intervention (Lipina and Colombo, 2009; D’Angiulli et al., 2012; Lipina and Posner, 2012). This also implies: (a) the support of methodological innovations in the analysis of neural connectivity for studies that compare different intervention contexts (e.g., home, school, community), its mediators, and the potential requirements for the intervention designs (e.g, Jolles and Crone, 2012; Lipina and Posner, 2012); and (b) the generation of alternative methodologies aimed at overcoming limits in sample size, timing of longitudinal designs, and levels of analysis (Gianaros and Hackman, 2013).

Finally, we find it important to improve the knowledge on the conceptualization of childhood poverty in terms of how children experience deprivation, and the generation of innovative ways to operationalize it in suitable terms for neuroscientific approaches (Lipina et al., 2011). This is especially important since the current neuroscientific evidence on developmental patterns has contributed to our understanding of poverty as a phenomenon much more complex and dynamic than the definitions proposed by other social and human scientific disciplines.

## Conflict of interest statement

The authors declare that the research was conducted in the absence of any commercial or financial relationships that could be construed as a potential conflict of interest.
